# Cytochrome P450-Catalyzed Hydroxylation of Δ8-Tetrahydrocannabinol and Implications for Pharmacogenetics

**DOI:** 10.3390/pharmaceutics18091149

**Published:** 2026-09-11

**Authors:** Mengqi Zhao, Philip Lazarus

**Affiliations:** Division of Molecular Biosciences, Department of Pharmaceutical Sciences, School of Pharmacy and Pharmaceutical Sciences, University at Buffalo, 160 Hayes Rd., Buffalo, NY 14215, USA; mzhao34@buffalo.edu

**Keywords:** cannabinoid, THC, CYP450, CYP2C9, CYP2C19

## Abstract

**Introduction:** The use of Δ8-tetrahydrocannabinol (Δ8-THC) has become increasingly prevalent in the United States, but its metabolic pathways remain poorly characterized. This study aimed to identify the key enzymes responsible for the 11′-hydroxylation of Δ8-THC, its primary metabolic pathway, and assess the effect of metabolizing enzyme genotype on this activity. **Results:** The intrinsic clearance of recombinant (r) CYP2C9 was >6200−fold higher than that observed for rCYP2C19, the only other tested CYP450 with detectable activity against Δ8-THC. The $K_{m,u}$ of rCYP2C9 (0.013 ± 0.0032 µM) was comparable to that observed in human intestinal microsomes (0.0076 ± 0.00067 µM), but it was substantially lower than that observed for human liver microsomes (0.73 ± 0.065 µM). Inhibition of 11-OH-Δ8-THC formation in rCYP-overexpressing microsomes was observed when using CYP probe inhibitors (sulfaphenazole for CYP2C9 and tranylcypromine for CYP2C19). The intrinsic clearance was markedly reduced in CYP2C9 variant microsomes, with 20− to 60−fold decreases observed for CYP2C9*2 (59 ± 7.9 µL·min^−1^·mg^−1^ protein), CYP2C9*3 (34 ± 3.6 µL·min^−1^·mg^−1^ protein), CYP2C9*8 (96 ± 13 µL·min^−1^·mg^−1^ protein), and CYP2C9*9 (34 ± 11 µL·min^−1^·mg^−1^ protein) as compared to wild-type CYP2C9*1 (6028 ± 266 µL·min^−1^·mg^−1^ protein). **Conclusions:** Given that the expression of CYP2C9 is much higher than that of CYP2C19 in both human intestine and liver, these data suggest that CYP2C9 is the primary enzyme responsible for intestinal 11′-hydroxylation of Δ8-THC and that other untested CYPs may be important in hepatic Δ8-THC hydroxylation. In addition, the reduced 11-OH-Δ8-THC formation activity observed with CYP2C9 variants (*2, *3, *8, *9) suggests interindividual variability in Δ8-THC disposition.

## 1. Significance Statement

This study represents the first in vitro characterization of CYP2C9 as the primary enzyme mediating intestinal 11′-hydroxylation of Δ8-THC and reveals that this activity is significantly modified and affected by the CYP2C9 genotype, suggesting potential interindividual variability in Δ8-THC disposition.

## 2. Background

**Figure 1:**
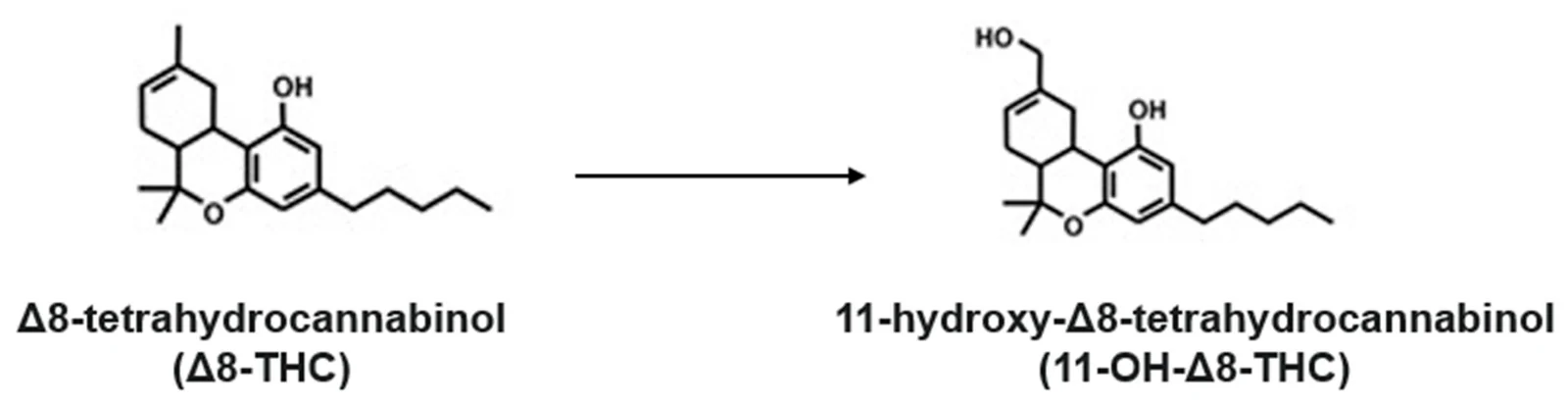
Schematic illustration of the enzymatic 11′-hydroxylation of Δ8-tetrahydrocannabinol (Δ8-THC) catalyzed by CYP2C9 and CYP2C19, leading to the formation of 11-hydroxy-Δ8-THC (11-OH-Δ8-THC).

Cannabis is one of the most widely used natural products worldwide and has been employed for centuries for both medicinal and recreational purposes [1]. Δ8-Tetrahydrocannabinol (THC) is a minor naturally occurring cannabinoid whose use has increased substantially in recent years, particularly in regions where the more psychoactive Δ9-THC remains legally restricted [2,3]. A recent U.S. survey reported that approximately 11% of 12th-grade students used Δ8-THC in the past year [4]. Despite its increasing use, the safety, pharmacology, and pharmacokinetics of Δ8-THC remain poorly understood [5,6]. Structurally, Δ8-THC is an isomer of the more psychoactive Δ9-THC, differing only in the position of a double bond within the cyclohexene ring [7]. Recent studies have shown that Δ8-THC may pose a risk for adverse events while also exhibiting potential therapeutic effects. Similar to other cannabinoids, including Δ9-THC and cannabidiol (CBD), Δ8-THC can modulate xenobiotic metabolism [8,9,10]. In vitro studies have demonstrated that Δ8-THC and its active metabolite, 11-OH-Δ8-THC, reversibly inhibit CYP2C9-mediated metabolism of warfarin and diclofenac as well as CYP3A4-mediated metabolism of midazolam, with static modeling predicting a high potential for clinically relevant drug–drug interactions [11]. Meanwhile, Δ8-THC irreversibly inhibits nicotine 5′-oxidation to cotinine, the primary pathway of nicotine clearance [12]. This inhibition may prolong systemic nicotine exposure, and it potentially influences smoking behavior [13,14]. Therefore, understanding the efficiency of Δ8-THC biotransformation to 11-OH-Δ8-THC (see Figure 1) is critical for comprehensively evaluating both its therapeutic potential as a smoking cessation aid and the potential risk of unintended drug–drug interactions (DDIs).

The metabolism and pharmacokinetics of other important cannabinoids, including cannabidiol (CBD) and Δ9-THC, have been well characterized [15,16]. CBD is primarily metabolized by CYP2C19 and CYP3A4 to form 7-hydroxy-CBD (7-OH-CBD), which is further oxidized to 7-carboxy-CBD, which is subsequently glucuronidated [17,18]. Δ9-THC undergoes hydroxylation primarily by CYP2C9, with minor contributions from CYP2C19, to form the active metabolite 11-hydroxy-Δ9-THC, which is further oxidized to the inactive metabolite 11-nor-9-carboxy-Δ9-THC [19]. The pharmacokinetics of Δ8-THC have been less extensively investigated, with available evidence suggesting that, similar to that observed for Δ9-THC, 11′-hydroxylation represents the primary metabolic pathway of Δ8-THC [20,21]. A clinical study reported that following a 20 mg oral dose, Δ8-THC reaches a similar time to peak concentration ($t_{max}$) as Δ9-THC, but with a higher maximum plasma concentration ($C_{max}$). Meanwhile, the formation of 11-hydroxy-Δ8-THC (11-OH-Δ8-THC) occurs later and at a lower $C_{max}$ as compared with that of Δ9-THC [22]. These findings suggest that Δ8-THC may be metabolized more slowly than Δ9-THC. While CYPs 2C9 and 2C19 were implicated in 11-OH-Δ8-THC formation [23], direct kinetic data remains limited, hindering the further development of PBPK models and reliable translational predictions for Δ8-THC-mediated DDIs and optimal dosing strategies.

Hepatic metabolism is the primary determinant of drug clearance and oral bioavailability, with intestinal metabolism serving as an additional barrier to systemic exposure [24,25]. CYP450 enzymes play a central role in Phase I metabolism in both liver and intestine [26,27], and major hepatic CYP enzymes—including CYP1A2, 2B6, 2C8, 2C9, 2C19, 2D6, 2E1, and 3A4—account for the metabolism of most clinically used drugs [28]. The present study aimed to identify the major cytochrome P450 enzymes responsible for the 11′-hydroxylation of Δ8-THC, characterize the associated enzyme kinetics, and assess the effects of metabolizing enzyme genotypes on this activity.

## 3. Methods

**Chemicals and reagents.** Δ8-THC, 11-OH-Δ8-THC, and the deuterated internal standard 11-OH-Δ8-THC-d_3_ were obtained from Cayman Chemical (Ann Arbor, MI, USA). The NADPH-regenerating system was purchased from Corning (Bedford, MA, USA). LC-MS-grade solvents, microcentrifuge tubes, and bicinchoninic acid (BCA) assay kits were acquired from Fisher Scientific (Waltham, MA, USA). All other chemicals used were of analytical grade or higher. The anti-V5 tag monoclonal antibody conjugated to horseradish peroxidase (HRP) (Catalog No. R96125) and the SuperSignal™ Femto Maximum Sensitivity Substrate were purchased from Novex, Fisher Scientific (Waltham, MA, USA). The anti-calnexin polyclonal rabbit antibody (Catalog No. 2433) and the HRP-linked anti-rabbit IgG antibody (Catalog No. 7044S) were purchased from Cell Signaling Technology (Danvers, MA, USA).

**Enzyme sources.** Pooled human liver microsomes (HLMs; mixed genders, n = 50 donors) and human intestinal microsomes (HIMs; mixed genders, n = 50 donors) were obtained from BioiVT (Lenexa, KS, USA). Microsomes from HEK293 cells individually overexpressing recombinant CYP450s 1A2, 2B6, 2C8, 2C9, 2C19, 2E1, 2D6, 3A4, and CYP2C9 *2, *3, *8, and *9 (Arg144Cys, Ile359Leu, Arg150His, and His251Arg, respectively) were prepared as previously described with all enzymes containing an N-terminal V5 tag for relative quantification analysis [29]. Protein concentrations for all microsomal samples were determined using the bicinchoninic acid (BCA) assay. The relative expression of the recombinant CYP450 enzymes was assessed by Western blot analysis [10]. Briefly, 20 µg of microsomal protein was separated on 10% SDS–polyacrylamide gels and transferred to polyvinylidene difluoride (PVDF) membranes using standard protocols. Membranes were blocked with 5% nonfat milk and incubated with an HRP-conjugated mouse monoclonal anti-V5 tag antibody (1:1000 dilution in 5% nonfat milk). To verify equal protein loading across lanes, membranes were also probed with an anti-calnexin polyclonal rabbit antibody (1:1000 dilution in 5% nonfat milk), followed by incubation with an HRP-linked anti-rabbit IgG secondary antibody for 1 h at room temperature. Protein bands were visualized using the SuperSignal™ Femto Maximum Sensitivity Substrate, and densitometric analysis was performed using ImageJ software ver. 1.54 (National Institutes of Health, Bethesda, MD, USA) [29]. Relative expression factors (REFs) were determined by normalizing the V5-HRP band intensities to calnexin, with the expression level of CYP2C19 set as the reference (1.0; see Supplemental Figure S1).

**Enzyme activity assays.** Our incubation procedure follows a widely used in vitro CYP activity assay protocol [17,30]. Due to its limited aqueous solubility, Δ8-THC was dissolved in an aqueous solution containing 3% methanol, which was selected to minimize solvent-related effects on CYP enzyme activity [31], and added to a 30 µL reaction assay containing 3 mM MgCl_2_, 100 mM potassium phosphate buffer (pH 7.4), and either recombinant P450 microsomes (50 µg), HIM (20 µg), or HLM (20 µg) in a total volume of 30 µL. Reactions were pre-warmed at 37 °C for 3 min and initiated by the addition of an NADPH-regenerating system (1.3 mM NADP, 3.3 mM glucose 6-phosphate, and 0.4 U/mL glucose 6-phosphate dehydrogenase). Incubations were carried out at 37 °C for 15 min with HLM and HIM and for 30 or 120 min with recombinant P450 microsomes. Reactions were terminated by the addition of 30 µL of ice-cold acetonitrile containing 0.2 µg/mL 11-OH-Δ8-THC-d_3_, followed by vortexing and centrifugation at 17,000× *g* for 15 min. Approximately 30 µL of the supernatant was transferred to UPLC–MS/MS vials, and 4 µL was then injected into the ultra-high-performance liquid chromatography (UHPLC)–tandem mass spectrometry system for analysis. To reduce nonspecific binding of cannabinoids, all incubations were performed in low-binding 0.6 mL centrifuge tubes. All experiments were performed in triplicate [32].

**Quantification of 11-OH-Δ8-THC.** To measure 11-OH-Δ8-THC levels, reaction supernatants were injected into a Waters Acquity UHPLC system coupled to a tandem Xevo TQD (triple quadrupole) mass spectrometer (Waters, Milford, MA, USA). Chromatographic separation was achieved on an Acquity UPLC BEH C_18_ column (2.1 × 100 mm, 1.7 µm) maintained at 40 °C, with a mobile phase consisting of (**A**) water containing 10 mM ammonium acetate and (**B**) acetonitrile with 0.1% formic acid. A 10 min gradient was applied as follows: 95% A for 2 min, a linear ramp to 95% B over 4 min, a 2 min hold at 95% B, and a 2 min re-equilibration at 95% A, at a flow rate of 0.4 mL/min. Detection was performed in positive electrospray ionization mode (ESI^+^) using multiple reaction monitoring (MRM) with the following mass-to-charge (m/z) transitions: Δ8-THC, 315.1 → 135.1; 11-OH-Δ8-THC, 331.2 → 193.1; and 11-OH-Δ8-THC-d_3_, 334.1 → 196.1. Metabolite formation was quantified with a standard curve generated from serial dilutions (0.019–5 µg/mL) of 11-OH-Δ8-THC reference standard, with 11-OH-Δ8-THC-d_3_ serving as the internal standard. The calibration curves exhibited strong linearity, with an r^2^ value of 0.9993. Data were processed using TargetLynx software (Waters) and further analyzed in Microsoft Excel and GraphPad Prism version 10.0 (GraphPad Software, San Diego, CA, USA).

**Kinetic analysis of 11-OH-Δ8-THC formation in HLM, HIM, and recombinant CYP450 microsomes.** The enzyme kinetic parameters (Δ8-THC concentration at half-maximal velocity [$K_{m}$] or [$S_{50}$], and maximum 11-OH-Δ8-THC formation rate [$V_{max}$]) for the 11′-hydroxylation of Δ8-THC in HLM, HIM, and recombinant P450 microsomes were determined using at least seven Δ8-THC concentrations ranging from 0.1 to 100 μM to fully characterize the enzyme kinetic profile [33]. Three independent experiments were performed to characterize all kinetic data. Three different kinetic models were fitted to the in vitro data (11-OH-Δ8-THC formation rates vs. Δ8-THC concentrations) by nonlinear regression analysis, including Michaelis–Menten (Equation (1)), allosteric sigmoidal (Hill) (Equation (2)), and substrate inhibition (Equation (3)), using GraphPad Prism version 10.0 [29,34,35,36]. In addition to using Eadie–Hofstee plots to guide the identification of appropriate enzyme kinetic models, F-tests were performed in GraphPad Prism to compare the goodness of fit of different kinetic models. The best-fit model, as determined by the F-test and goodness-of-fit parameters, including R^2^, was selected for estimation of the enzyme kinetic parameters.

(1) $$v=\frac{V_{max}\times S}{K_{m}+S}$$

(2) $$v=\frac{V_{max}\times S^{h}}{S_{50}^{h}+S^{h}}$$

(3) $$v=\frac{V_{max}\times S}{K_{m}+S\times (1+\frac{S}{K_{i}})}$$

v indicates the metabolite (11-OH-Δ8-THC) formation rate; $V_{max}$ is the maximum rate of metabolite formation; S is the substrate concentration; $K_{m}$ is the Michaelis–Menten constant; $S_{50}$ is the substrate concentration resulting in 50% of $V_{max}$ (analogous to $K_{m}$ in the Michaelis–Menten model); and h is the Hill coefficient. When h > 1.0, the binding of the first ligand increases the affinity of the protein for subsequent ligands, creating a steep sigmoidal (S-shaped) binding curve. When h = 1.0, the binding of a ligand is independent. The probability of ligand binding does not change, regardless of how many other ligands are already bound. $K_{i}$ is the inhibition constant, describing the equilibrium dissociation constant for the enzyme-inhibitor complex and can be calculated using Equation (3) [37]. $K_{m}$, $S_{50}$, and $K_{i}$ values were corrected for abundant nonspecific binding of Δ8-THC to the labware and protein, multiplying by the unbound fraction ($f_{u}$); (Equations (4)–(6)). Given their highly similar chemical structures and properties, the $f_{u}$ of Δ9-THC was used (0.043 in recombinant P450 microsomes and 0.051 in HLM; 0.051 was used as a surrogate $f_{u}$ for incubations with HIM) as reported previously [8].

(4) $$K_{m,u}=K_{m}\times f_{u}$$

(5) $$S_{50,u}=S_{50}\times f_{u}$$

(6) $$K_{i,u}=K_{i}\times f_{u}$$

Intrinsic clearance [${CL}_{int}$] (for the Michaelis–Menten model) and maximal clearance [${CL}_{max}$] (for the Hill model) for 11′-hydroxylation of Δ8-THC in HLM, HIM, and recombinant P450 microsomes were calculated as described in Equation (7) and Equation (8), respectively [37].

(7) $${CL}_{int}=\frac{V_{max}}{K_{m,u}}$$

(8) $${CL}_{max}=\frac{V_{max}}{S_{50,u}}\times \frac{h-1}{h\times {\left(h-1\right)}^{\frac{1}{h}}}$$

where h is the Hill coefficient, which describes the extent of cooperativity of ligand binding to multi-subunit proteins or enzymes.

The levels of 11-OH-Δ8-THC formation were corrected by the REF of individual CYP450s in either HLM or HLM as determined by Western blot analysis described above (Equation (9) for different CYP450 enzymes and Equation (10) for different CYP2C9 variants).

(9) $${Formation}_{11-OH-\Delta 8-THC}\;=\;{Formation}_{11-OH-\Delta 8-THC,detected}\;\times REF$$

(10) $${Formation}_{11-OH-\Delta 8-THC}\;=\;\frac{{Formation}_{11-OH-\Delta 8-THC,detected}}{REF}$$

**CYP450 inhibition studies.** To verify the involvement of major hepatic CYP enzymes in the 11′-hydroxylation of Δ8-THC, chemical inhibition assays were performed using recombinant human P450 microsomes. Probe inhibitor (1 or 10 µM; sulfaphenazole for CYP2C9 and tranylcypromine for CYP2C19) was added to 30 µL reaction assays containing Δ8-THC (10 µM) or probe control substrates (10 µM diclofenac for CYP2C9, or 1 µM omeprazole for CYP2C19), 3 mM MgCl_2_, 100 mM potassium phosphate buffer (pH 7.4), and 50 µg recombinant CYP2C9 or CYP2C19 microsomal protein [11]. Probe substrates were used at concentrations approximating their known $K_{m}$’s for their respective CYP enzyme to minimize off-target interactions [38,39]. Reaction mixtures were pre-incubated at 37 °C for 3 min before being initiated with the NADPH-regenerating system. Assays were incubated at 37 °C for 15 min, terminated by the addition of 30 µL ice-cold acetonitrile containing the internal standard (11-OH-Δ8-THC-d_3_) and processed as described above. Detection of diclofenac and omeprazole metabolites and %Activity calculations were performed as described previously [40].

**Statistical analysis.** One-way ANOVA (analysis of variance) was used to determine if there were statistically significant differences between the means of three or more independent groups, while individual comparisons were assessed using a two-sided Student’s *t*-test, with all tests considered statistically significant at *p* < 0.05. Statistical analyses were performed for all comparisons, with all experiments performed in triplicate. Statistical analyses were performed using GraphPad Prism 10.0 (GraphPad Software, San Diego, CA, USA).

## 4. Results

**Figure 2:**
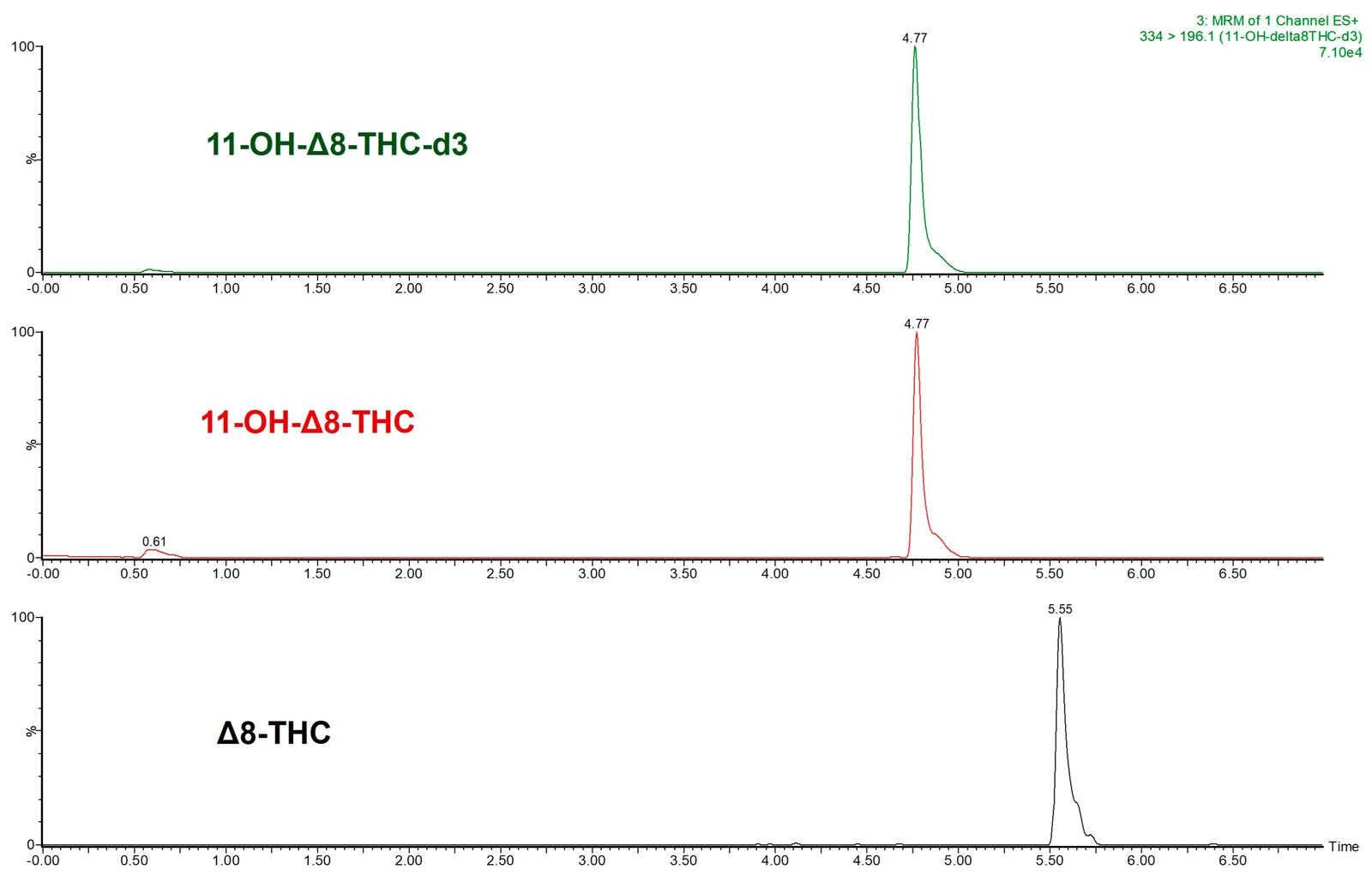
Representative UHPLC−MS/MS chromatograms of Δ8-tetrahydrocannabinol (Δ8-THC, black), 11-hydroxy-Δ8-THC (11-OH-Δ8-THC, red), and the internal standard 11-OH-Δ8-THC−d_3_ (green). The X-axis is the elution time, and the Y-axis is the signal response in mass spectrometry analysis.

**Figure 3:**
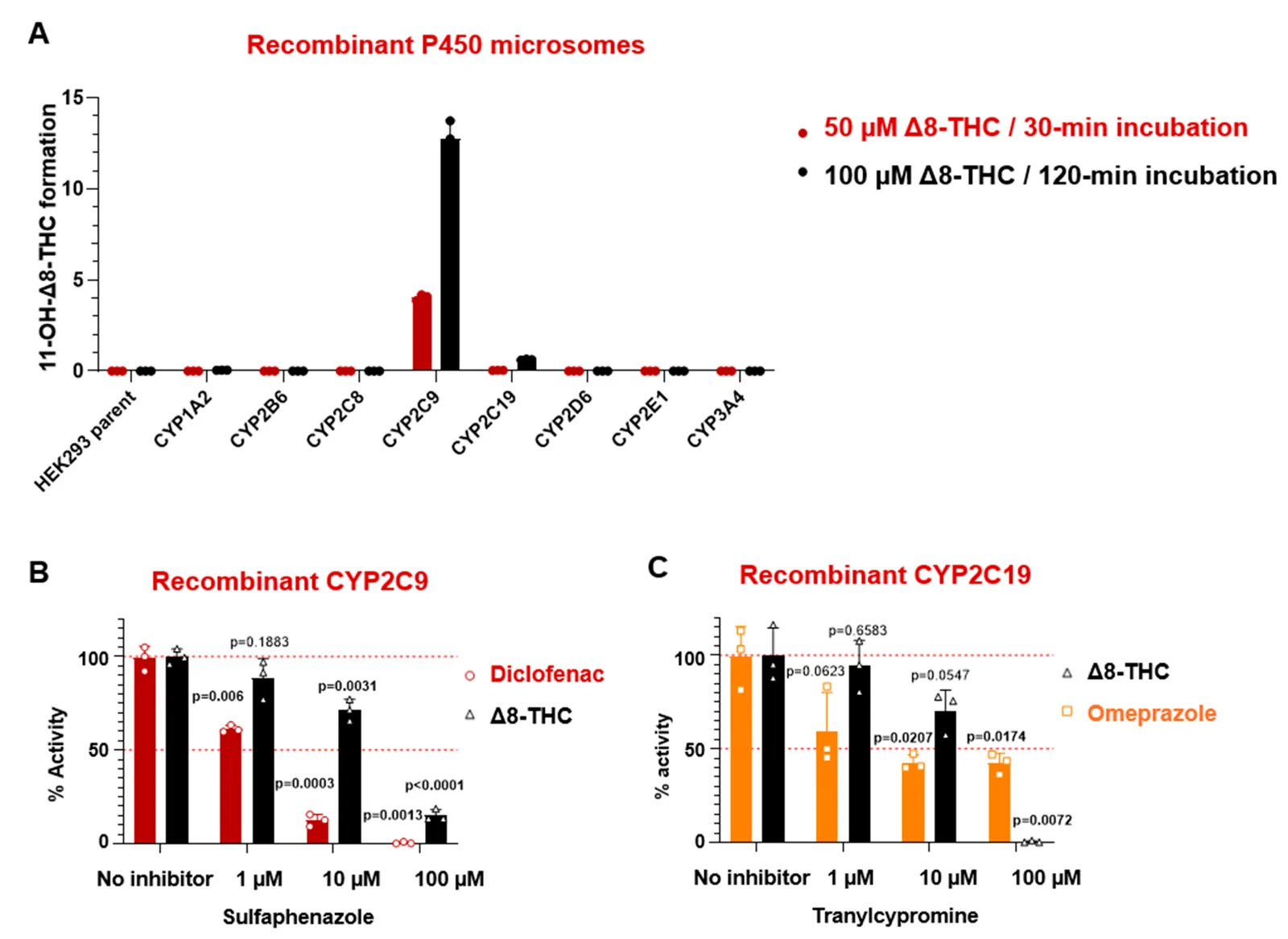
Screening of CYP enzymes responsible for the 11′−hydroxylation of Δ8-THC: (**A**) Major hepatic cytochrome P450 enzymes were incubated with Δ8-THC for 30 min (50 µM, red) or 120 min (100 µM, black); enzyme activities are indicated by levels of 11-OH-Δ8-THC formation. (**B**) Chemical inhibition of recombinant CYP2C9 microsomes incubated with diclofenac (red) or Δ8-THC (black) by 0, 1, 10, or 100 µM sulfaphenazole. (**C**) Chemical inhibition of recombinant CYP2C19 microsomes incubated with omeprazole (orange) or Δ8-THC (black) by 0, 1, 10, or 100 µM tranylcypromine. Comparisons were assessed using a two-sided Student’s *t*-test, with all tests considered statistically significant at *p* < 0.05 (significant values shown in bold). All experiments were performed in triplicate. Statistical analysis was conducted using GraphPad Prism 10.0 (GraphPad Software, San Diego, CA, USA).

As shown in a representative chromatogram of a reference sample mixture containing 0.1 µg/mL of each compound, UHPLC-MS/MS peaks for Δ8-THC (retention time = 5.55 min), 11-OH-Δ8-THC (retention time = 4.77 min), and 11-OH-Δ8-THC-d_3_ (retention time = 4.77 min) were clearly identified (Figure 2). To determine the major hepatic cytochrome P450 enzymes responsible for the 11′-hydroxylation of Δ8-THC, enzyme activity assays were conducted by incubating 50 µM or 100 µM Δ8-THC with microsomes from recombinant CYP-overexpressing HEK293 cells (or parental untransfected HEK293 cells as a negative control) for 30 or 120 min, respectively. Recombinant CYP2C9 microsomes produced the highest levels of 11-OH-Δ8-THC, while recombinant CYP2C19 microsomes yielded substantially lower amounts (Figure 3A). The formation of 11-OH-Δ8-THC by CYP2C9 was approximately 10−fold higher than that observed for CYP2C19 under both incubation conditions. No detectable formation of 11-OH-Δ8-THC was observed in microsomes from other recombinant CYP enzymes (i.e., CYPs 1A2, 2B6, 2C8, 2D6, 2E1, and 3A4) or from parental HEK293 cells.

The roles of CYP2C9 and CYP2C19 were validated through chemical inhibition studies using probe inhibitors in incubations with HLM–sulfaphenazole for CYP2C9 and tranylcypromine for CYP2C19. 11-OH-Δ8-THC formation was significantly reduced in incubations containing recombinant CYP2C9-overexpressing microsomes and either 10 µM (27% reduction, *p* = 0.0031) or 100 µM (85% reduction, *p* < 0.0001) sulfaphenazole. Sulfaphenazole also significantly inhibited recombinant CYP2C9 activity against its probe substrate, diclofenac, in a concentration-dependent manner (40% at 1 µM sulfaphenazole, *p* = 0.006; 85% at 10 µM sulfaphenazole, *p* = 0.0003; and 99% at 100 µM sulfaphenazole, *p* = 0.0013; Figure 3B). A similar pattern was observed with tranylcypromine as the probe inhibitor for recombinant CYP2C19 microsomes, with a significant reduction in 11-OH-Δ8-THC formation observed at 100 µM Δ8-THC (99%, *p* = 0.0072; Figure 3C).

**Figure 4:**
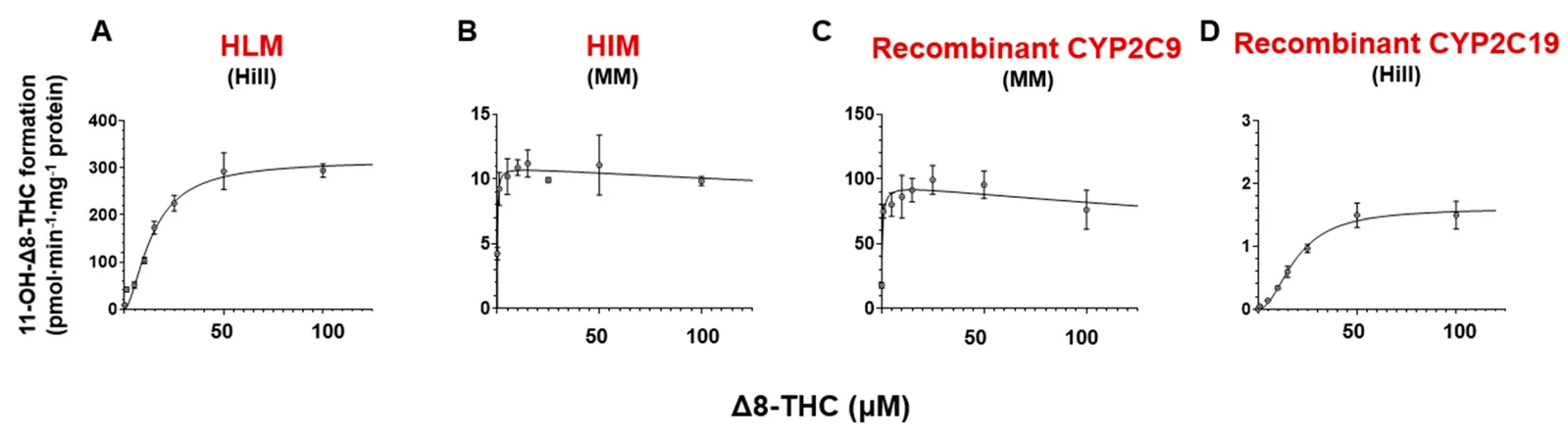
Representative CYP enzyme kinetic curves of 11′−hydroxylation of Δ8-THC: (**A**) Human liver microsomes (HLMs) fitted with the Hill model, (**B**) human intestine microsomes (HIMs) fitted with the Michaelis–Menten (MM) model, (**C**) recombinant CYP2C9 microsomes fitted with the MM model, and (**D**) recombinant CYP2C19 microsomes fitted with the Hill model. Each point represents the mean ± standard deviation of three individual experiments.

To confirm the contributions from CYP2C9 and CYP2C19 in Δ8-THC 11′-hydroxylation, the kinetics of this pathway were further characterized using microsomes from HEK293 cells overexpressing recombinant CYP2C9 or CYP2C19, human liver microsomes (HLMs), or human intestinal microsomes (HIMs). CYP activity assay data were fitted to Michaelis–Menten and Hill models to estimate $V_{max}$ and $K_{m,u}$. Nonlinear regression of metabolite formation versus substrate concentration revealed similar kinetic behavior between HIM and recombinant CYP2C9 microsomes (Figure 4). In both systems, the best fit was achieved using the Michaelis–Menten model (no significant difference after F-test, larger R^2^ values), with the reaction velocity increasing rapidly and then reaching a plateau at lower concentrations (below 1 µM). Different kinetic profiles were observed for HLM and recombinant CYP2C19 microsomes, with the Hill model providing a slightly better fit than the Michaelis–Menten model and both showing a more gradual increase in metabolite formation, approaching a plateau only at higher substrate concentrations (~100 µM). The Hill coefficients were 2.0 ± 0.11 for recombinant CYP2C19 and 1.6 ± 0.20 for HLM, with values greater than 1 suggesting potential cooperative substrate binding or multiple active-site interactions [29,41]. The Eadie–Hofstee plot for HLM exhibited two distinct slopes (R_1_^2^ = 0.99; R_2_^2^ = 0.61), suggesting the presence of two active-site interactions, which can be explained by the observation that more than one CYP enzyme is involved in hepatic 11-OH-Δ8-THC formation (Supplemental Figure S2). In contrast, single linear regression of the Eadie–Hofstee plots for HIM (R^2^ = 0.80), recombinant CYP2C9 (R^2^ = 0.89), and CYP2C19 microsomes (R^2^ = 0.69) revealed a single slope, suggesting a single active-site interaction.

Kinetic analysis of the 11′-hydroxylation of Δ8-THC with recombinant CYP-overexpressing microsomes showed much smaller $K_{m,u}$ and higher *V_max_* values for CYP2C9 as compared to CYP2C19, resulting in an intrinsic clearance for recombinant CYP2C9 (1985 ± 339 µL·min^−1^·mg^−1^ protein) that was > 2000−fold that observed for CYP2C19 (0.97 ± 0.093 µL·min^−1^·mg^−1^ protein; Table 1). In addition, the $K_{m,u}$ of CYP2C9 (0.013 ± 0.0032 µM) was similar to that observed for HIM (0.0076 ± 0.00067 µM), again consistent with the high relative levels of expression of CYP2C9 vs. CYP2C19 in the human intestine and the possibility that CYP2C9 is the major enzyme involved in the 11′-hydroxylation of Δ8-THC in the human intestine [42]. The intrinsic clearance for HIM (1405 ± 68 µL·min^−1^·mg^−1^ protein) was 6.2−fold higher than that observed for HLM (225 ± 23 µL·min^−1^·mg^−1^ protein), a pattern again consistent with the high relative expression of CYP2C9 vs. CYP2C19 in human intestine [43].

While the $K_{m,u}$ for HLM (0.73 ± 0.065 µM) was much higher than that observed for recombinant CYP2C9 microsomes, it matched well with that observed for recombinant CYP2C19 microsomes. However, this contrasts with the higher levels of expression of CYP2C9 vs. CYP2C19 in the liver, suggesting that other CYP enzymes in addition to CYP2C9 and perhaps CYP2C19 may also play an important role in the hepatic 11′-hydroxylation of Δ8-THC. This is consistent with the fact that no inhibition of 11′-hydroxylation of Δ8-THC was observed in HLM with sulfaphenazole or tranylcypromine, probe inhibitors for CYP2C9 or CYP2C19, respectively (Supplemental Figure S3).

**Figure 5:**
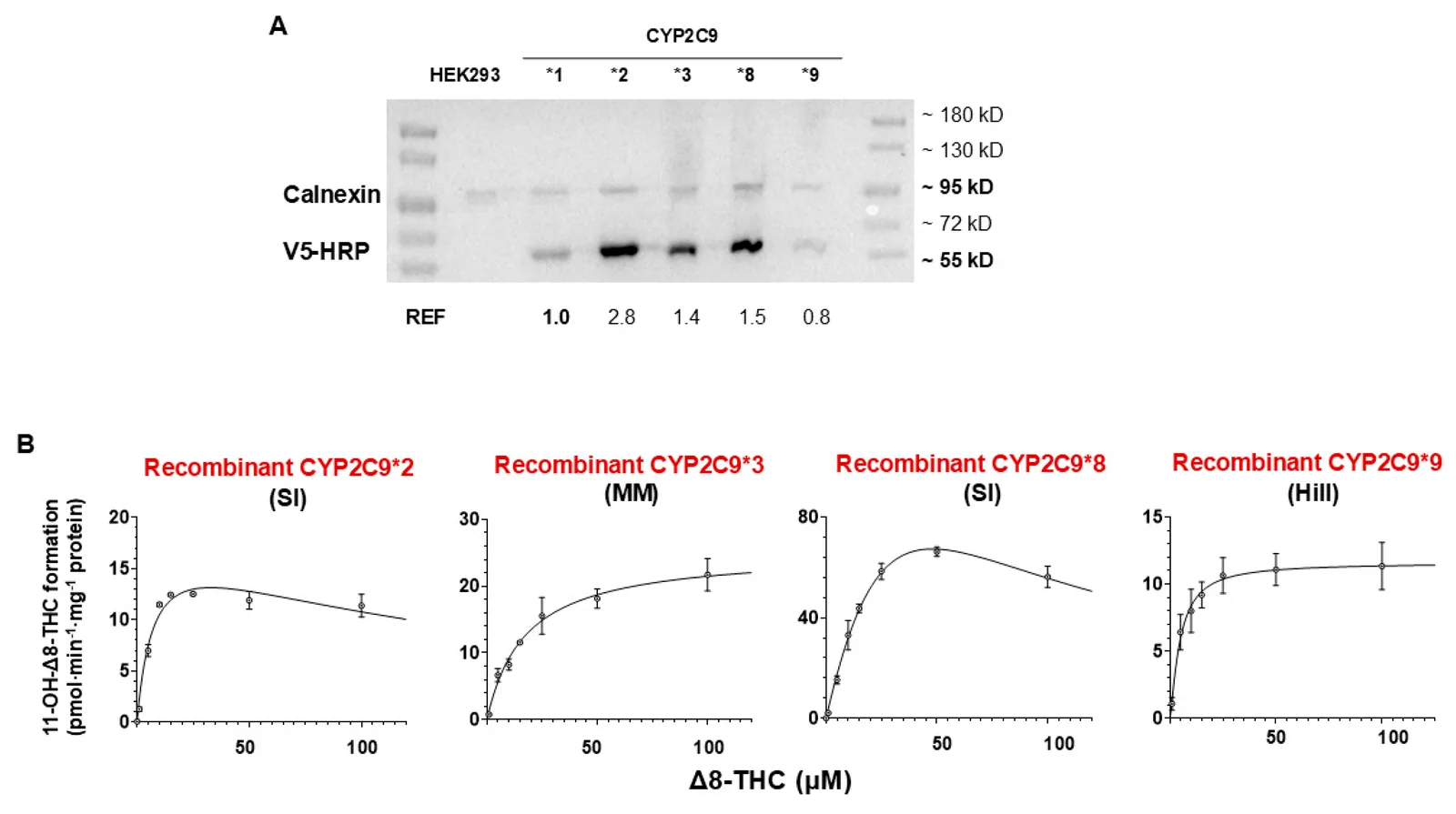
The impact of CYP2C9 variants on the 11′-hydroxylation of Δ8-THC: (**A**) Western blot analysis showing the expression of V5-tagged recombinant CYP2C9 variants in overexpressing HEK293 microsomes. REF indicates their relative expression factors, with the wild-type CYP2C9*1 designated as 1.0. (**B**) Representative enzyme kinetic curves of the 11′-hydroylation of Δ8-THC for recombinant CYP2C9*2 fitted with the substrate inhibition (SI) model, recombinant CYP2C9*3 fitted with the Michaelis–Menten (MM) model, recombinant CYP2C9*8 fitted with the SI model, and recombinant CYP2C9*9 fitted with the Hill model.

CYP2C9 is a polymorphic enzyme with several variants that exhibit a high minor allele frequency (MAF; >0.02) in at least one major population group (European, African, East Asian, or South Asian; see Supplemental Table S1) and contain non-synonomous single-nucleotide polymorphisms (the CYP2C9 *2, *3, *8, and *9 variants) [44,45,46,47]. The kinetics of 11-OH-Δ8-THC formation for CYP2C9 variants were assessed using recombinant microsomes from HEK293 cells overexpressing each CYP2C9 variant, with 11-OH-Δ8-THC formation normalized based on their relative levels of expression in the different CYP2C9 variant-overexpressing cell lines with CYP2C9*1 as the reference (REF = 1.0; see Figure 5A). Activity assays were conducted using at least seven concentrations of Δ8-THC (0.1–100 µM), and the resulting data were fitted to multiple kinetic models (Michaelis–Menten, substrate inhibition, and Hill) to estimate the $V_{max}$ and $K_{m,u}$ (or $S_{50,u}$). Unlike that observed for microsomes from wild-type CYP2C9*1-overexpressing cells, microsomes from both the CYP2C9 *2 and *8 variants exhibited substrate inhibition (Figure 5B), a pattern also observed in Eadie–Hofstee plots (Supplemental Figure S2), where the data points formed a semi-circular distribution. The estimated $K_{i},u$ from the substrate inhibition model were 6.7 ± 3.2 µM for recombinant CYP2C9*2 and 1.5 ± 0.68 µM for recombinant CYP2C9*8. The metabolic kinetics of the CYP2C9 *3 and *9 variants were better described by Michaelis–Menten and Hill models, respectively (Figure 5B). The Hill coefficient was 1.3 ± 0.14 for recombinant CYP2C9*9, with a value greater than 1 suggesting potential cooperative substrate binding or multiple active-site interactions [29,41]. Single linear regression of Eadie–Hofstee plots (Supplemental Figure S2) showed a single slope for the recombinant CYP2C9 *3 (R^2^ = 0.78) and *9 microsomes (R^2^ = 0.96), suggesting Michaelis–Menten or Hill models with a single active-site interaction and potential cooperative substrate binding for CYP2C9*9.

Significant (*p* < 0.05; ANOVA) reductions in intrinsic clearance (${CL}_{int}$ or ${CL}_{max}$) were observed for all tested CYP2C9 variants (Table 2, Supplemental Table S2). Compared to microsomes from wild-type CYP2C9*1-overexpressing cells (1985 ± 339 µL∙min^−1^∙mg^−1^ protein), CYP2C9*2, CYP2C9*3, CYP2C9*8, and CYP2C9*9 microsomes exhibited 34− (59 ± 7.9 µL∙min^−1^∙mg^−1^ protein; *p* = 0.010), 58− (34 ± 3.6 µL∙min^−1^∙mg^−1^ protein; *p* = 0.0099), 21− (96 ± 13 µL∙min^−1^∙mg^−1^ protein; *p* = 0.011), and 58− (34 ± 11 µL∙min^−1^∙mg^−1^; *p* = 0.0099) fold significant decreases in intrinsic clearance, respectively. The significant decreases in catalytic efficiency of CYP2C9 variants were mainly caused by increases in $K_{m,u}$ (or $S_{50,u}$) (Table 2, Supplemental Table S2). Specifically, microsomes from the CYP2C9*2, CYP2C9*3, and CYP2C9*9 variants displayed $K_{m,u}$ values (0.35 ± 0.10 µM, *p* = 0.028; 0.76 ± 0.074 µM, *p* = 0.0032; and 0.21 ± 0.033 µM, *p* = 0.0091; respectively) that were 13 − 45 fold significantly lower than that observed for CYP2C9*1 (0.013 ± 0.0032 µM). CYP2C9*8 microsomes exhibited an even greater increase in $K_{m,u}$ (3.2 ± 1.3 µM), which was 246−fold (*p* = 0.051) greater than that observed for CYP2C9*1 microsomes. Compared to CYP2C9*1 microsomes (26 ± 2.9 pmol∙min^−1^∙mg^−1^ protein), no significant change in the $V_{max}$ value of CYP2C9 *2 and *3 was observed. A significant 2−fold reduction and an 11−fold increase in $V_{max}$ was observed for CYP2C9*9 (12 ± 1.3 pmol∙min^−1^∙mg^−1^, *p* = 0.032) and CYP2C9*8 microsomes (292 ± 85 pmol∙min^−1^∙mg^−1^**,** *p* = 0.0059), respectively, as compared to CYP2C9*1 microsomes. Together, these findings suggest that the Arg144Cys, Ile359Leu, Arg150His, and His251Arg amino acid substitutions corresponding to the *2, *3, *8, and *9 variants, respectively, significantly impair the catalytic efficiency of CYP2C9 toward, mainly by altering the binding efficiency of Δ8-THC to CYP2C9.

## 5. Discussion

Previous studies have implicated CYPs 2C9 and 2C19 as the enzymes involved in Δ8-THC metabolism [23]. Results from the present study suggest that CYP2C9 appears to be the major enzyme involved in the 11′-hydroxylation of Δ8-THC in the human intestine. While CYP2C19 also showed detectable levels of 11-OH-Δ8-THC formation activity, the intrinsic clearance for CYP2C19 was over 2000−fold lower than that observed for CYP2C9. In addition, the $K_{m,u}$ observed for CYP2C9 (0.013 μM) was similar to that observed for HIM (0.0076 μM), further implicating CYP2C9 as the main intestinal enzyme involved in 11-OH-Δ8-THC formation. Given that CYP2C19 exhibits between 3.8− and 11−fold lower levels of expression as compared to CYP2C9 in various regions of the intestine [42], this suggests that CYP2C19 plays a minor role at best in intestinal 11-OH-Δ8-THC formation.

Interestingly, the $K_{m,u}$ observed for CYP2C9 was approximately 56−fold lower than that observed for HLM (0.73 μM). Since CYP2C19 microsomes exhibited a $K_{m,u}$ (0.83 μM) similar to that observed for HLM, this enzyme may be playing a role in the hepatic clearance of Δ8-THC. However, the expression of CYP2C19 was shown to represent only 1% of total hepatic CYP protein and is 27−fold lower than that observed for CYP2C9 [42,43], which exhibited a much higher intrinsic clearance for Δ8-THC than CYP2C19. In addition, probe inhibitors of CYPs 2C9 and 2C19 exhibited little inhibition of HLM-mediated 11-OH-Δ8-THC formation. Taken together, this implicates other CYP enzymes that were not screened in the present study in hepatic 11-OH-Δ8-THC formation. As the primary organ responsible for xenobiotic metabolism, the liver contains a diverse array of phase I drug-metabolizing enzymes, including not only many CYP enzymes but also other enzyme families that utilize NADPH as a cofactor and may catalyze similar metabolic reactions [32,48]. A comprehensive screening of all potentially involved enzymes should therefore be performed for Δ8-THC metabolism.

The metabolic efficiency of CYP2C9 is significantly reduced (by 21− to 58−fold) in recombinant microsomes expressing the CYP2C9 *2 (Arg144Cys), *3 (Ile359Leu), *8 (Arg150His), and *9 (His251Arg) variants. These findings underscore the critical role of these residues in the 11′-hydroxylation of Δ8-THC and highlight the need for clinical studies to evaluate the impact of CYP2C9 genetic variants on the pharmacokinetics of Δ8-THC.

The $K_{m}$ of CYP2C9 for the 11′-hydroxylation of Δ8-THC (0.31 ± 0.0074 µM) observed in the present study is lower than that observed for Δ9-THC (2.13 ± 0.41 µM) in previous studies [49]. However, the $V_{max}$ for CYP2C9 for 11-OH-Δ8-THC formation (26 nmol·min^−1^·nmol^−1^ protein) observed in the present study was comparable to that observed in another study (19.2 nmol·min^−1^·nmol^−1^ protein) [23]. While differences in kinetics could be due to the recombinant systems used in the different studies (i.e., overexpressing HEK293 cells vs. baculosomes), the data suggest a higher catalytic affinity for CYP2C9 for Δ8-THC vs. Δ9-THC.

The Δ8-THC $K_{m}$ in HLM (14 ± 1.3 µM) observed in the present study was substantially higher than that observed for Δ9-THC ($K_{m}$ = 0.0014 ± 0.2 µM) in one study [19], and marginally higher than that observed for Δ9-THC ($K_{m}$ = 5.2 ± 2.0 µM) in another study [49]. Interestingly, the $V_{max}$ for Δ8-THC observed for HLM (318 ± 20 pmol·min^−1^·mg^−1^ protein) in the present study was comparable to that reported previously for Δ9-THC (521 ± 53 pmol·min^−1^·mg^−1^ protein) in previous studies [19,23]. This suggests that the liver may exhibit a lower catalytic affinity for Δ8-THC than Δ9-THC. Unfortunately, no previous studies have been performed examining Δ9-THC in HIM.

The impact of CYP2C9 genetic variants on the metabolism of Δ8-THC, Δ9-THC, and other drugs varies significantly and was more pronounced for Δ8-THC in the present study as compared to that observed for Δ9-THC in previous studies. For example, the CYP2C9*2 variant exhibited a 27−fold increase in $K_{m}$ or $S_{50}$ for the 11′-hydroxylation of Δ8-THC as compared to wild-type CYP2C9*1 in the present study, which is markedly higher than the 5−fold increase observed for Δ9-THC [49]. In terms of intrinsic clearance, a previous study reported that CYP2C9*2 exhibited a 2.5−fold reduction in Δ9-THC 11′-hydroxylation as compared to wild-type CYP2C9*1 [49], whereas a 34−fold reduction was observed for Δ8-THC in the present study. A similar pattern is observed for the CYP2C9*3 variant, with 58−fold increases in both the $K_{m}$ and intrinsic clearance observed for 11-OH-Δ8-THC formation as compared to the wild-type CYP2C9*1 in the present study vs. 3−fold increases observed for Δ9-THC in previous studies [49]. Clinically, individuals with the CYP2C9 (*3/*3) genotype exhibited a 2−fold higher systemic exposure (e.g., $C_{max}$) for Δ9-THC as compared to those with the wild-type genotype (CYP2C9 *1/*1) [50]. Therefore, larger effects would be predicted for Δ8-THC. These differences indicate that the functional effects of CYP2C9 genetic variation are highly substrate-dependent and that clinical investigations are required to better examine the potential impact of CYP2C9 variants on Δ8-THC metabolism and pharmacokinetics.

Some minor limitations should be noted. The aqueous solubility of Δ8-THC is very limited [51], with visible precipitation observed at concentrations as low as 1 mM, restricting our ability to evaluate metabolic kinetics at higher substrate concentrations (>100 µM), which may have contributed to uncertainty in the estimation of $K_{i}$ values derived from substrate inhibition models. Also, the fraction unbound (*f_u_*) value for Δ8-THC was not characterized in this or previous studies, with the (*f_u_*) values observed for Δ9-THC used as a surrogate. This concern is mitigated to a large extent by the similar chemical structures, inhibition profiles, and kinetics of the two THC isomers [11]. Previous studies have shown that cannabidiol (CBD), which has a lower logP (~6.0) than either Δ8-THC and Δ9-THC, also exhibits a similar unbound fraction to Δ9-THC in vitro, with both Δ9-THC and CBD showing < 10% unbound [52]. Finally, as described above, other CYP enzymes not examined in the present study may be active against Δ8-THC in the liver. A larger assessment of additional CYP enzymes will be necessary to further characterize the hepatic clearance of Δ8-THC.

## 6. Conclusions

In summary, of the eight major CYP enzymes examined in this study, CYP2C9 appears to be the primary enzyme involved in the 11′-hydroxylation of Δ8-THC in the intestine. While CYPs 2C9 and 2C19 may be involved in hepatic 11-OH-Δ8-THC formation, it appears that other enzymes may also be involved. In addition, genetic variations in CYP2C9 appear to exert a significant effect on the metabolism of Δ8-THC, an effect that may be greater than that observed for Δ9-THC. This is especially pertinent in the intestine and first-pass metabolism for orally administered THC, where CYP2C9 plays a major role in 11-OH-Δ8-THC formation. Future in vitro studies in hepatocytes as well as human pharmacokinetic studies are needed to confirm the predicted impact of CYP2C9 genetic variants on Δ8-THC metabolism.

## Figures and Tables

**Table 1:** Enzyme kinetic parameters for Δ8-THC hydroxylation by recombinant P450 microsomes, HLM and HIM.

Enzyme Source	$K_{m}$ or $S_{50}$ (μM) ^b^	$K_{m,u}$ or $S_{50,u}$ (μM) ^b^	$V_{max}$ ^c^ (pmol∙min^−1^∙mg^−1^ Total Protein)	${CL}_{int}$ or ${CL}_{max}$ ^d^ (µL·min^−1^·mg^−1^ Total Protein)	Model
Rec CYP2C9 ^a^	0.31 ± 0.0074	0.013 ± 0.0032	26 ± 2.9	1985 ± 339	MM
Rec CYP2C19	19 ± 2.4	0.83 ± 0.10	1.6 ± 0.23	0.97 ± 0.093	Hill
HLM	14 ± 1.3	0.73 ± 0.065	318 ± 20	225 ± 23	Hill
HIM	0.15 ± 0.013	0.0076 ± 0.00067	11 ± 0.91	1405 ± 68	MM

^a^ Rec, recombinant microsomes from recombinant P450 overexpressing-HEK293 cells; HLM, human liver microsomes; HIM, human intestinal microsomes; MM, Michaelis Menton model. ^b^ $S_{50}$ is the substrate concentration resulting in 50% of $V_{max}$ in the Hill model (analogous to $K_{m}$ in the Michaelis-Menten model). $K_{m,u}$ or $S_{50,u}$ is the corrected $K_{m}$ or $S_{50}$ calculated by multiplying the unbound fraction ($f_{u}$) in the tube using $f_{u}$ for Δ9-THC (0.051 in HLM, and 0.043 in recombinant P450 microsomes) as a surrogate for Δ8-THC. ^c^ $V_{max}$ values in recombinant P450 microsomes were normalized by relative expression levels as shown in Supplemental Figure S1 (recombinant CYP2C9 = 3.5, recombinant CYP2C19 = 1.0). ^d^ ${CL}_{int}$ was calculated as $\frac{V_{max}}{K_{m,u}}$ in the MM model, ${CL}_{max}$ was calculated as $\frac{V_{max}}{S_{50,u}}\;\times \;\frac{h-1}{h\times {\left(h-1\right)}^{\frac{1}{h}}}$ in the Hill model. The Hill coefficients were 2.0 ± 0.11 for recombinant CYP2C19, and 1.6 ± 0.20 for HLM.

**Table 2:** Enzyme kinetic parameters for Δ8-THC hydroxylation by recombinant CYP2C9 variants microsomes.

Rec CYP2C9 ^a^ Microsomes	$K_{m}$ or $S_{50}$ (μM) ^b^	$K_{m,u}$ or $S_{50,u}$ (μM) ^b^	$V_{max}$ ^c^ (pmol∙min^−1^∙mg^−1^ Protein)	${CL}_{int}$ or ${CL}_{max}$ ^d^ (µL·min^−1^·mg^−1^ Protein)	Model
*1	0.31 ± 0.0074	0.013 ± 0.0032	26 ± 2.9	1985 ± 339	MM ^e^
*2	8.0 ± 2.3	0.35 ± 0.10	20 ± 2.6	59 ± 7.9	SI ^e^
*3	18 ± 1.7	0.76 ± 0.074	25 ± 1.9	34 ± 3.6	MM
*8	73 ± 30	3.2 ± 1.3	292 ± 85	96 ± 13	SI ^e^
*9	4.9 ± 0.78	0.21 ± 0.033	12 ± 1.3	34 ± 11	Hill

^a^ Rec, recombinant microsomes from recombinant P450 overexpressing-HEK293 cells; MM, Michaelis Mention model; SI, substrate inhibition model. ^b^ $S_{50}$ is the substrate concentration resulting in 50% of $V_{max}$ in the Hill model (analogous to the $K_{m}$ in the Michaelis-Menten model). $K_{m,u}$ or $S_{50,u}$ calculated by multiplying the unbound fraction ($f_{u}$) in the tube using $f_{u}$ for Δ9-THC (0.051 in HLM, and 0.043 in recombinant P450 microsomes) as a surrogate for Δ8-THC. ^c^ $V_{max}$ values in recombinant P450 microsomes were normalized by relative expression levels as shown in Figure 5. ^d^ ${CL}_{int}$ was calculated as $\frac{V_{max}}{K_{m,u}}$ in the MM and SI models; ${CL}_{max}$ was calculated as $\frac{V_{max}}{S_{50,u}}\;\times \;\frac{h-1}{h\times {\left(h-1\right)}^{\frac{1}{h}}}$ in the Hill model. The Hill coefficient was 1.3 ± 0.1 for recombinant CYP2C9*9. ^e^ For the substrate inhibition model, the estimated unbound $K_{i}$ values ($K_{i},u)$, corrected using the $f_{u}$ for Δ9-THC as described above, were 6.7 ± 3.2 µM for recombinant CYP2C9*2 and 1.5 ± 0.68 µM for recombinant CYP2C9*8.

## Data Availability

All data supporting these findings are contained within the paper.

## Supplementary Materials

The following supporting information can be downloaded at: https://www.mdpi.com/article/10.3390/pharmaceutics18091149/s1, Figure S1: Expression of V5-tagged recombinant P450 enzymes in overexpressing HEK293 microsomes. Figure S2: Chemical inhibition of 11’-hydroxylation of Δ8-THC in human liver microsomes, and human intestine microsomes. Figure S3: Eadie-Hofstee plots for the 11’-hydroxylation of Δ8-THC in human liver microsomes, human intestine microsomes, recombinant CYP2C9 microsomes, recombinant CYP2C19 microsomes, recombinant CYP2C9*2 microsomes, recombinant CYP2C9*3 microsomes, recombinant CYP2C9*8 microsomes, and recombinant CYP2C9*9 microsomes; Table S1: Minor allele frequencies of CYP2C9 *2, *3, *8, and *9 in different populations. Table S2: Impact of CYP2C9 *2, *3, *8, and *9 variants on Δ8-THC 11’-hydroxylation.

## Abbreviations

CBD, cannabidiol; Δ9-THC, Δ9-tetrahydrocannabinol; Δ8-THC, Δ8-tetrahydrocannabinol; 11-OH-Δ8-tetrahydrocannabinol, 11-hydroxy-Δ8-tetrahydrocannabinol; Δ8-THC-COOH, 11-nor-Δ8-tetrahydrocannabinol-9-carboxylic acid; FDA, Food and Drug Administration; HLMs, human liver microsomes; HIMs, human intestinal microsomes; $f_{u}$, unbound fraction of cannabinoid in in vitro assays; HEK, human embryonic kidney; LC, liquid chromatography; MS, mass spectrometry; MS/MS, tandem MS; CYP450, cytochrome P450; UHPLC, ultra-high-pressure liquid chromatography.
