# Supplementary material for: Cytochrome P450-Catalyzed Hydroxylation of Δ8-Tetrahydrocannabinol and Implications for Pharmacogenetics
Source: Pharmaceutics. 2026 Sep 11;18(9):1149. doi: 10.3390/pharmaceutics18091149 (PMC13610851; doi:10.3390/pharmaceutics18091149)
Supplement: Supplementary file 1 [file pharmaceutics-18-01149-s001.zip › pharmaceutics-4518230-supplementary-tables.pdf]

Supplemental Tables

Supplemental Table S1. Minor allele frequencies of CYP2C9 *2, *3, *8, and *9 in different populations.

**CYP2C9 Variant**	**Genetic Impact**	**Minor Allele Frequency (MAF)**			
		**European**	**African**	**Eastern Asian**	**South Asian**
*2	Arg144Cys	**0.13** [a]	**0.02**	< 0.01	**0.05**
*3	Ile359Leu	**0.07**	0.01	**0.03**	**0.11**
*8	Arg150His	< 0.01	**0.06**	< 0.01	< 0.01
*9	His251Arg	< 0.01	**0.08**	< 0.01	< 0.01

[a] MAF > 0.02 are bolded.

Supplemental Table S2. Impact of CYP2C9 *2, *3, *8, and *9 variants on Δ8-THC 11'-hydroxylation.

		Fold-difference as compared to CYP2C9*1 [a]		
Rec CYP2C9 microsomes [b]	Amino acid change	$K_{m,u}$ or $S_{50,u}$ (μM) [c]	V_{max} (pmol·min^{-1}·mg^{-1} protein)	CL_{int} or CL_{max} (□L·min^{-1}·mg^{-1} protein)
*2	Arg144Cys	**27 (0.028)**	1.3 (0.0527)	**34 (0.010)**
*3	Ile359Leu	**58 (0.0032)**	1.0 (0.78)	**58 (0.0099)**
*8	Arg150His	246 (0.051)	**11 (0.032)**	**21 (0.011)**
*9	His251Arg	**16 (0.0091)**	**2.2 (0.0059)**	**58 (0.0099)**

[a] Comparisons were assessed using a two-sided Students t-test, with all tests considered statistically significant at P < 0.05 (bolded). Values in parenthesis are the P-values vs CYP2C9*1.

[b] Rec, microsomes from recombinant P450 overexpressing HEK293 cells.

[c] S_{50} is the substrate concentration resulting in 50% of V_{max} (analogous to K_m in the Michaelis-Menten model). $K_{m,u}$ or $S_{50,u}$ is the corrected K_m or S_{50} by multiplying the unbound fraction (f_u) of Δ9-THC in the tube (0.051 in HLM, and 0.043 in recombinant P450 microsomes).
